# Superior sagittal sinus thrombosis in the course of mixed phenotype acute leukaemia treated with acute lymphoblastic leukaemia-like therapy—a case report

**DOI:** 10.1186/s12959-023-00561-9

**Published:** 2023-11-16

**Authors:** Wojciech Lizurej, Łukasz Mazurkiewicz, Michał Kowalski, Sylwia Szydłowska, Michał Wyrzykowski, Krzysztof Lewandowski

**Affiliations:** 1 Department of Hematology and Bone Marrow Transplantation, Poznan University of Medical Sciences, Poznan, Poland; 2 Department of Diagnostic Imaging, Poznan University of Medical Sciences, Poznan, Poland

**Keywords:** Mixed phenotype acute leukaemia, PEG-asparaginase, Thrombosis, LMWH, Antithrombin, Treatment, Case report

## Abstract

Mixed phenotype acute leukaemia (MPAL) is associated with worse overall survival, compared with other acute leukaemias in adults. Lack of clear treatment guidelines makes the therapy challenging. ALL-like induction and consolidation treatment followed by allo-HSCT is the preferred first-line treatment. We present a case of a 36-year-old woman diagnosed with MPAL (EGIL Myelo/B) with KMT2A rearrangement, treated with the PALG-ALL-7 (including PEG-asparaginase) protocol. On day 25 after the induction therapy initiation, numbness of limbs and dizziness were observed. Therefore, the imaging studies (CT and MRI) were performed and a diagnosis of thrombosis of superior sagittal sinus of the brain was established. Routinely performed blood coagulation tests showed prolonged APTT and PT, decreased antithrombin III activity and decreased free protein S concentration. LMWH treatment and substitutional therapy with antithrombin III were started, which resulted in a significant reduction in the thrombosis associated symptoms and improvement of the neurological status after 3 days. After induction and consolidation therapy, the patient obtained complete haematological remission and negative measurable residual disease. Six months after the diagnosis, allo-HSCT was successfully performed. During the 4 months follow-up, the patient remained MRD negative and thrombotic symptoms free. To the best of our knowledge, our communication has been the first report of such complication in an MPAL patient treated with PEG-asparaginase containing protocol in adults. We recommend increased vigilance in patients manifesting any mild neurological symptoms and early decision about the MRI study performance.

**Supplementary Information:**

The online version contains supplementary material available at 10.1186/s12959-023-00561-9.

## Background

Mixed phenotype acute leukaemia (MPAL) is a subtype of acute leukaemia with coexistent presence of phenotypic features specific for both myeloid and lymphoid lineages. It accounts for 2–5% of all acute leukaemias with worse overall survival, compared with other disease types in adults [1, 2]. It is classified as biphenotic MPAL, where single blast population demonstrates features of different lineages (myeloid, B-cell or/and T-cell), as well as bilineage MPAL, where distinct blast populations express different lineage markers independently [1].

Current World Health Organization (WHO) criteria divide MPAL into three genomic categories, depending on the presence of KMT2A rearrangement, BCR-ABL fusion or other defined genetic alterations. Other MPAL cases are classified as immunophenotypically defined and include B/myeloid (59%), T/myeloid (35%), or T/B (4%), T/B/myeloid (2%) phenotype [3–5]. Furthermore, MPAL can also be diagnosed according to the European Group for Immunological Classification of Leukaemia (EGIL), where the diagnosis of MPAL requires scoring at least two points in two distinct lineages (Supplementary Table 1) [4, 6].

Lack of clear guidelines concerning the appropriate way of treatment result in challenges in curing MPAL [4, 7]. The latest meta-analysis of 1300 patients including small series and case reports of patients with MPAL diagnosed based on EGIL or WHO criteria, revealed that acute lymphoblastic leukaemia like (ALL)-like therapy induction is superior compared with acute myeloid leukaemia (AML)-like induction treatment, due to significantly higher complete haematologic response (CHR) rates and a two-fold reduction in the mortality risk [8]. Nowadays, ALL-like induction treatment followed by allogeneic haematopoietic stem cell transplantation (allo-HSCT) is recommended in patients with MPAL as the first-line of treatment [4].

## Case presentation

Herein, we present a case of a 36-year-old woman diagnosed with MPAL, with no past medical history, with an unusual thrombotic complication after induction therapy. The patient was admitted to the Department of Haematology and Bone Marrow Transplantation in October 2022, due to progressive lymphadenopathy (enlarged circumferential lymph nodes) and bleeding symptoms (increased bruising). Laboratory tests, including multicolour flow cytometry (MFC) of the bone marrow cells confirmed that 88% of cells expressed CD13 0.0%, CD33 90%, CD34 73%, CD117 0.0%, HLA DR 75%, CD38 100%, CD31 100%, CD36 0,0%, CD14 0,0%, CD11b 0.0%, CD11c 0.0%, CD64 57%, CD163 0.0%, CD18 61%, CD56 0.0%, CD16 0.0%, CD19 57%, CD10 0.0%, CD20 0.0%, CD22 0.0%, CD3 0.0%, CD5 0.0%, CD4 0.0%, CD8 0.0%, CD7 0.0%, CD2 0.0%, CD1a 0.0%, CD66b 0.0%, CD66c 0.0%, CD65 32%, CD15 80%, CD123 100%, cytCD68 0.0%, MPO 0.0%, TdT 0.0%, cytCD3 0.0%, cytCD79a 28%, cytCD22 0.0%, cyt.IgM 0.0%, CLL-1 68%, CRLF2 0.0%, surface kappa 0.0%, pow lambda 0.0%//CD45 mid, FSC mid, SSC low (EGIL classification: myeloid line − 3 points, B-lymphoid line − 3 points). Molecular analysis with RT-PCR did not confirm BCR::ABL gene, PML::RARA gene, FLT3 D835 mutation, FLT3-ITD, and NPM1 mutation. FISH study of the bone marrow cells was positive for KMT2A-r (Vysis®, IL, USA) and negative for BCR::ABL fusion (Cytocell®, Cambridge, UK).

**Fig. 1:**
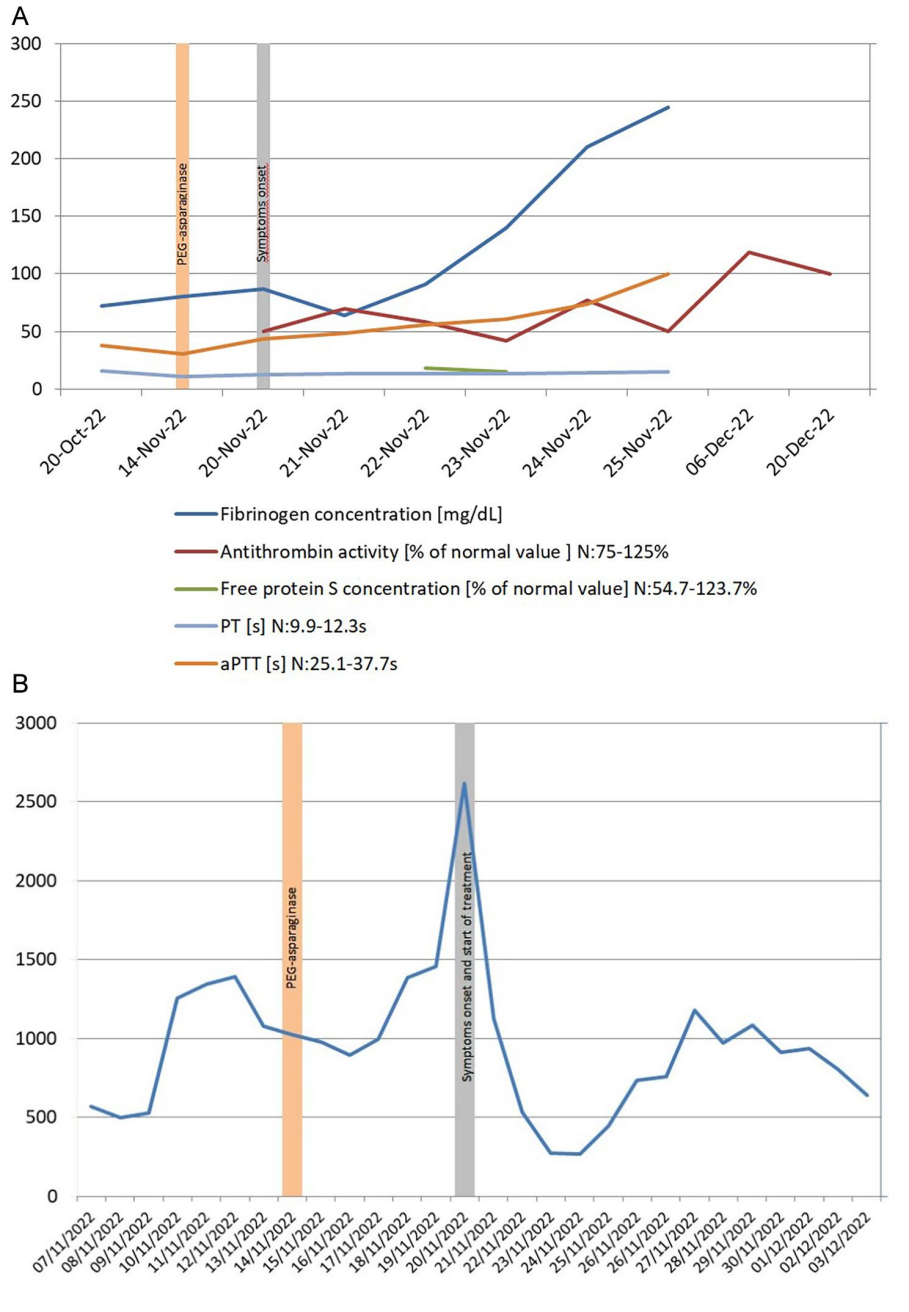
Assessment of fibrinogen concentration, antithrombin activity, free protein S plasma content, PT, aPTT and D-dimers during induction therapy. Graphical presentation of haematological parameters (fibrinogen concentration, antithrombin activity, free protein S plasma content, PT and aPTT) of our patient before, during and after the clinical manifestation of the disease (**A**). D-dimers plasma level during induction L-asparaginase, containing chemotherapy application in the presented case (**B**)

**Fig. 2:**
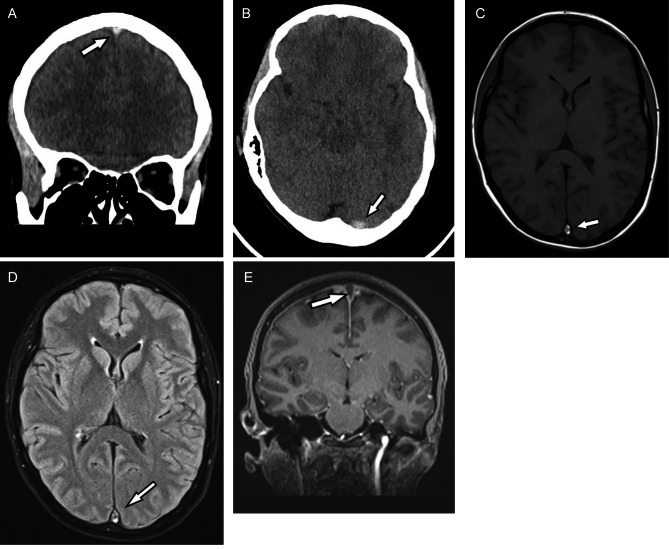
**A**-**E**. The images reveal characteristic findings of cerebral venous sinus thrombosis in a 36-year-old female patient with superior sagittal sinus thrombosis. Coronal non-enhanced CT scan (**A**) and axial non-enhanced CT scan (**B**) show areas of abnormal hyper attenuation in the superior sagittal sinus (**A**) and the proximal part of left transverse sinus that are consistent with thrombosis. Axial T1 weighted MRI image (**C**) and axial fluid-attenuated inversion recovery (FLAIR) image (**D**) shows thrombus as an area of increased signal intensity in the superior sagittal sinus. Coronal contrast-enhanced MR reveals thrombus as filling defects in the superior sagittal sinus (**E**)

CT scans of the chest and abdomen revealed enlarged lymph nodes in the mediastinum, right axillar fossa, along major blood vessels, liver and spleen enlargement. Cytopathological examination of central nervous fluid (CSF) did not confirm leptomeningeal involvement of malignant cells. Finally, the diagnosis of MPAL (EGIL Myelo/B) with the KMT2A rearrangement within the 11q23 was established. Therapy according to the Polish Adult Leukaemia Group ALL-7 protocol (PALG ALL-7) was started in October 2022 [daunorubicine 83.5 mg—day 1, 8, 15, 22, vincristine 2.0 mg—day 1, 8, 15, 22, cytarabine 40.0 mg—day 13, 27, PEG-asparaginase (*Spectrile®*) 3.340 UI—day 20, methotrexate 15.0 mg—day 13, 27] [9]. On day 26 after the induction therapy initiation (6 days after PEG-asparaginase infusion), numbness of limbs and dizziness were noted. Routine blood coagulation tests showed prolonged APTT = 55.9s (N: 25.1-37.7s) and PT = 13.6s (N: 9.9-12.3s), and a drop in antithrombin III activity (54% [N: 83–128%]) and free protein S concentration (18.2% [N: 54.7-123.7%]). Also fibrinogen concentration in the blood was significantly reduced (Fig. 1A). Moreover, a significant increase in the D-dimers concentration was noted (Fig. 1B). CT and MRI studies revealed the thrombosis of venous sinuses of the brain (superior sagittal sinus and a proximal part of left transverse sinus) (Fig. 2A-E).

Therefore, substitutional therapy with antithrombin III (*Anbinex*®) and low molecular weight heparin (LMWH) at a dose 1 mg/kg b.w. subcutaneously—6000 IU, 60 mg (LMWH, Clexane®) was started immediately. The therapy resulted in significant reduction of the thrombosis associated symptoms and improvement of the neurological status after 3 days. Fresh frozen plasma or cryoprecipitate was administrated only if fibrinogen concentration was < 100 mg/dL. The evaluation of the response to induction therapy confirmed CHR. MFC assessment of the bone marrow cells on day 35 after induction therapy showed the presence of measurable residual disease (MRD) – 0.002%. Consolidation therapy with intermediate doses of cytarabine was given without any important complications (MRD = 0.0%). A repeated CT scan was performed in March 2023, revealing no signs of thrombosis.

Meanwhile, a fully matched family donor was identified. Allo-HSCT with CyTBI 12 Gy conditioning and graft versus host (GvHD) prophylaxis with thymoglobulin, cyclosporine, methotrexate was performed six months after the diagnosis in April 2023. Post-transplant disease monitoring revealed CHR, full donor chimerism and the absence of MRD and GvHD symptoms.

## Discussion and conclusions

The treatment of MPAL is challenging. According to recent literature data, the superiority of ALL-like therapy regiments (containing PEG-asparaginase) over AML-like therapy is suggested [7, 10–12].

However, it should be mentioned that the final therapy result depends on fully matched donor availability and patient MRD-negative status before allo-HSCT [7].

In humans, intravenous administration of L-asparaginase leads to a decrease in the serum asparagine level, due to a reduction in the synthesis of fibrinogen, plasminogen and anti-coagulant factors, as well, including antithrombin III, protein C, and protein S [13]. Its application may result in thrombosis and/or bleeding complications thereafter, due to the drug induced blood coagulation and natural anticoagulation system abnormalities [14]. Other PEG-asparaginase administration associated side effects include hypertriglyceridemia, hepatotoxicity and pancreatitis [15].

The frequency of thrombotic and bleeding complications associated with asparaginase treatment in ALL patients is age-dependent [16]. The occurrence of venous thromboembolic events (VTE) in patients with ALL treated with L-asparaginase containing regimens varies from 1 to 36%, depending on the treatment protocol, the inclusion/exclusion of asymptomatic thrombotic events, and whether the study was prospective or retrospective [17], where the incidence of VTE at the time of ALL diagnosis varies between 1.4 and 2.09% [18, 19]. The data obtained from clinical trials conducted at Dana-Farber Cancer Institute in the years 1991–2008, concerning patients with newly diagnosed ALL, revealed that 5% of paediatric patients, 34% of adult patients, and 42% of adults above 30 years of age suffered from VTE during intensive L-asparaginase therapy, and that the age was the only significant predictor of the thrombosis occurrence [16]. According to the available data, the use of less intensive L-asparaginase treatment regimens and employment of the antithrombin replacement therapy is associated with lower incidence of VTE events [20]. VTE occurs more frequently in adults treated with pegylated formulation of asparaginase (PEG-asparaginase) compared to L-asparaginase, despite appropriate prophylactic anticoagulation treatment [21, 22]. Underwood et al. revealed that if an initial VTE incident appeared in patients treated due to ALL, it happened usually during the induction phase (72%), followed by consolidation (11%) and delayed intensification (11%), rather then during maintenance therapy (6%). The cumulative incidence rate of thrombotic complication within 30 days after the administration of the initial dose of drug was 25%. However, it should be mentioned, that the median time between the previous asparaginase dose and the VTE episode occurrence was 14 days, where the range was 6–99 days [23].

Previous studies involving patients with ALL treated with L-asparaginase containing regimens found that objectively confirmed thrombosis occurred in 18.5% of patients during the induction phase, with a range between day 3 and day 12 after the L-asparaginase infusion [24]. In another study, 4.2% of patients presented thrombotic complications, which occurred between 5 and 15 days after the first dose of asparaginase, with a median onset on day 11 [25].

Thrombotic events in the course of PEG-asparaginase treatment are mostly venous and most often affect lower or upper extremities, the pulmonary system, or cerebral site [23, 26]. Data obtained from Dana-Farber Cancer Institute Consortium ALL protocols reveals that thrombotic events were localized most frequently in the venous circulation of the upper extremities or were central venous catheters related (36%). VTE disease was also localized in the lower extremities (19%) and the central nervous system (19%) [16].

In 15% of patients pulmonary embolism (PE) was documented [16]. In an observational study involving patients with ALL, the majority of pulmonary embolism incidents (78%) occurred during the L-asparaginase treatment phase. The complication was associated with a 30-day mortality rate of 9.4% [27]. Pulmonary embolism episode may influence the established leukaemia treatment [28]. Unexpectedly, according to the available data including 63% of patients who experienced PE during treatment, the total administered doses of L-asparaginase remained unchanged [27].

Overall occurrence of cerebral venous thrombosis (CVT) is estimated to be 1.3–1.6 per 100.000, with a mortality rate of 5–10% [29]. A study performed in Norway by Kristoffersen et al. showed slightly higher frequency of CVT (1.75 per 100.000 with a 30-day mortality rate at 3% and 1-year mortality rate at 6%) [30]. Couturier et al. report the prevalence of central nervous system thrombosis of 3.1%. It occurred within the range of 11 to 31 days, with a median of 18 days, in patients receiving L-asparaginase [31]. A previous systematic review emphasizes various mortality rates in acute phase of CVT ranging from 0 to 15.2%; mean 5.6% and the mean overall mortality at 9.4%, where underlying conditions, most often cancer, are responsible for half of these deaths [32]. In recent years, a significant reduction in the mortality rate among all the patients with CVT has been observed [33]. Ferro et al. distinguished predictors of death, such as male sex, age above 37 years, coma, cancer etc. [34]. Despite relatively common occurrence of CVT, its localization in the cavernous sinus is rarely observed [35].

Aldoss et al. observed that the cavernous sinus thrombosis appeared in 2 of 152 (1.3%) patients treated with regimens for ALL concerning L-asparaginase [26].

In the available literature, there is no data concerning the frequency of thrombotic complications in patients with MPAL treated with ALL-like therapy. To the best of our knowledge, our communication is the first report of such complication in MPAL patient treated with PEG-asparaginase containing protocol in adults. The available screening resources showed only one report by Wani et al. of a 13-year-old girl diagnosed with MPAL treated with induction therapy containing L-asparaginase 10,000 units, vincristine 2 mg, daunorubicin 40 mg who experienced a headache with an episode of focal seizure. The blood coagulation study results confirmed an abnormal prothrombin time (PT = 57% of normal), activated partial thromboplastin time (APTT = 62s) and prothrombin time (INR = 1.38). MRI enabled setting the diagnosis of dural sinus thrombosis. Therefore, treatment with LMWH and oral warfarin was introduced, with a significant improvement in the patient status after 5 days and full recovery from the symptoms thereafter [36].

Another very important issue is the use of PEG-asparaginase treatment monitoring not only for hypersensitivity symptoms occurrence, but also therapeutic effect [37].

Prophylaxis includes antithrombin level monitoring and infusion, while under the level of 50–60%. Short term administration of antithrombin may be considered. LMWH prophylaxis may be considered, too. The treatment of thrombotic episodes due to L-asparaginase treatment in MPAL patients should be based on therapeutic doses of LMWH. According to the available data, re-exposure to L-asparaginase when anticoagulant therapy with LMWH is given is safe [38, 39].

However, it should be stressed that therapeutic doses of LMWH may be safely used when platelet count is > 50 × 10^9^/L, while reduced doses should be used when platelet count is below this level [40]. Antithrombin III activity lower than or equal to 60% is considered a heparin resistance factor [41]. According to the PALG-ALL7 protocol guidelines, blood coagulation tests (APTT, antithrombin activity, fibrinogen concentration, INR) should be performed, before initiating L-asparaginase treatment (day 20), immediately before every L-asparaginase dose and one week post-treatment (day 34) [42]. Another important issue is early identification of thrombotic complication on the basis of regular monitoring of D-dimers concentration in the blood and patients’ clinical status [43]. The above mentioned strategy seems to be reasonable also in younger patients, which was confirmed by the positive outcome in our patient with MPAL experiencing dural venous sinus thrombosis at the age of 36.

In the presented case, despite the unusual localisation of thrombotic complication, superior sagittal sinus thrombosis was diagnosed early after the PEG-asparaginase administration. Therefore, we recommend increased vigilance in patients manifesting any mild neurological symptoms and early decision about the CT/MRI study performance. It may allow to avoid the fatal disease outcome due to undiagnosed thrombotic complication after PEG-asparaginase administration.

### Electronic supplementary material

Below is the link to the electronic supplementary material.

Supplementary Material 1

## Data Availability

Not applicable.

## List of abbreviations

MPAL: mixed phenotype acute leukaemia
WHO: World Health Organisation
MFC: multicolour flow cytometry
CSF: central nervous fluid
LMWH: low molecular weight heparin
MRD: measurable residual disease
CHR: complete haematological remission
GvHD: graft versus host disease
VTE: venous thromboembolic events
PE: pulmonary embolism

## References

[1] Weinberg OK, Arber DA (2010). Mixed-phenotype acute Leukemia: historical overview and a new definition. Leukemia.

[2] Khan M, Siddiqi R, Naqvi K (2018). An update on classification, genetics, and clinical approach to mixed phenotype acute Leukemia (MPAL). Ann Hematol.

[3] Khoury JD, Solary E, Abla O, Akkari Y, Alaggio R, Apperley JF (2022). The 5th edition of the World Health Organization Classification of Haematolymphoid Tumours: myeloid and Histiocytic/Dendritic Neoplasms. Leukemia.

[4] George BS, Yohannan B, Gonzalez A, Rios A (2022). Mixed-phenotype Acute Leukemia: clinical diagnosis and therapeutic strategies. Biomedicines.

[5] Mi X, Griffin G, Lee W, Patel S, Ohgami R, Ok CY (2018). Genomic and clinical characterization of B/T mixed phenotype acute Leukemia reveals recurrent features and T-ALL like mutations. Am J Hematol.

[6] Bene MC, Castoldi G, Knapp W, Ludwig WD, Matutes E, Orfao A (1995). Proposals for the immunological classification of acute leukemias. European Group for the Immunological Characterization of Leukemias (EGIL). Leukemia.

[7] Lazzarotto D, Tanasi I, Vitale A, Piccini M, Dargenio M, Giglio F (2023). Multicenter retrospective analysis of clinical outcome of adult patients with mixed-phenotype acute Leukemia treated with acute myeloid leukemia-like or acute lymphoblastic leukemia-like chemotherapy and impact of allogeneic stem cell transplantation: a campus ALL study. Ann Hematol.

[8] Maruffi M, Sposto R, Oberley MJ, Kysh L, Orgel E (2018). Therapy for children and adults with mixed phenotype acute Leukemia: a systematic review and meta-analysis. Leukemia.

[9] Polska Grupa ds. Leczenia Białaczek u Dorosłych. Protokół leczenia ostrej białaczki limfoblastycznej u dorosłych. Polish Adult Leukemia Group (PALG) guidelines for the treatment of ALL. [Internet]. 2022 [cited 2023 Jul 16]. Available from: www.palg.pl.

[10] Rasekh EO, Osman R, Ibraheem D, Madney Y, Radwan E, Gameel A (2021). Acute lymphoblastic leukemia-like treatment regimen provides better response in mixed phenotype acute Leukemia: a comparative study between adults and pediatric MPAL patients. Ann Hematol.

[11] Silverman LB, Supko JG, Stevenson KE, Woodward C, Vrooman LM, Neuberg DS (2010). Intravenous PEG-asparaginase during remission induction in children and adolescents with newly diagnosed acute lymphoblastic Leukemia. Blood.

[12] Béné MC, Porwit A (2022). Mixed Phenotype/Lineage Leukemia: has anything changed for 2021 on diagnosis, classification, and treatment?. Curr Oncol Rep.

[13] Hongo T, Okada S, Ohzeki T, Ohta H, Nishimura SI, Hamamoto K (2002). Low plasma levels of hemostatic proteins during the induction phase in children with acute lymphoblastic Leukemia: a retrospective study by the JACLS. Japan Association of Childhood Leukemia Study. Pediatr Int off J Jpn Pediatr Soc.

[14] Stock W, Douer D, DeAngelo DJ, Arellano M, Advani A, Damon L (2011). Prevention and management of asparaginase/pegasparaginase-associated toxicities in adults and older adolescents: recommendations of an expert panel. Leuk Lymphoma.

[15] Riley DO, Schlefman JM, Vitzthum Von Eckstaedt VHC, Morris AL, Keng MK, El Chaer F (2021). Pegaspargase in practice: minimizing toxicity, Maximizing Benefit. Curr Hematol Malig Rep.

[16] Grace RF, Dahlberg SE, Neuberg D, Sallan SE, Connors JM, Neufeld EJ (2011). The frequency and management of asparaginase-related Thrombosis in paediatric and adult patients with acute lymphoblastic Leukaemia treated on Dana-Farber Cancer Institute consortium protocols. Br J Haematol.

[17] Payne JH, Vora AJ (2007). Thrombosis and acute lymphoblastic Leukaemia. Br J Haematol.

[18] De Stefano V, Sorà F, Rossi E, Chiusolo P, Laurenti L, Fianchi L (2005). The risk of Thrombosis in patients with acute Leukemia: occurrence of Thrombosis at diagnosis and during treatment. J Thromb Haemost JTH.

[19] Ziegler S, Sperr WR, Knöbl P, Lehr S, Weltermann A, Jäger U (2005). Symptomatic venous thromboembolism in acute Leukemia. Incidence, risk factors, and impact on prognosis. Thromb Res.

[20] Hunault-Berger M, Chevallier P, Delain M, Bulabois CE, Bologna S, Bernard M (2008). Changes in antithrombin and fibrinogen levels during induction chemotherapy with L-asparaginase in adult patients with acute lymphoblastic Leukemia or lymphoblastic Lymphoma. Use of supportive coagulation therapy and clinical outcome: the CAPELAL study. Haematologica.

[21] Chen R, Atenafu EG, Seki J, Liu X, Chan S, Gupta V (2023). Venous thromboembolism incidence associated with pegylated asparaginase (ASP) compared to the native L-ASP: a retrospective analysis with an ASP-based protocol in adult patients with acute lymphoblastic Leukaemia. Br J Haematol.

[22] Hoelzer D, Bassan R, Dombret H, Fielding A, Ribera JM, Buske C (2016). Acute lymphoblastic Leukaemia in adult patients: ESMO Clinical Practice guidelines for diagnosis, treatment and follow-up. Ann Oncol off J Eur Soc Med Oncol.

[23] Underwood B, Zhao Q, Walker AR, Mims AS, Vasu S, Long M (2020). Incidence of venous Thrombosis after peg-asparaginase in adolescent and young adults with acute lymphoblastic Leukemia. Int J Hematol Oncol.

[24] Elliott MA, Wolf RC, Hook CC, Pruthi RK, Heit JA, Letendre LL (2004). Thromboembolism in adults with acute lymphoblastic Leukemia during induction with L-asparaginase-containing multi-agent regimens: incidence, risk factors, and possible role of antithrombin. Leuk Lymphoma.

[25] Gugliotta L, Mazzucconi MG, Leone G, Mattioli-Belmonte M, Defazio D, Annino L (1992). Incidence of thrombotic Complications in adult patients with acute lymphoblastic Leukaemia receiving L-asparaginase during induction therapy: a retrospective study. The GIMEMA Group. Eur J Haematol.

[26] Aldoss I, Douer D, Behrendt CE, Chaudhary P, Mohrbacher A, Vrona J (2016). Toxicity profile of repeated doses of PEG-asparaginase incorporated into a pediatric-type regimen for adult acute lymphoblastic Leukemia. Eur J Haematol.

[27] Tuckuviene R, Bjerg CL, Jonsson OG, Langstrom S, Rank CU, Ranta S (2020). Pulmonary Embolism in acute lymphoblastic Leukemia — an observational study of 1685 patients treated according to the NOPHO ALL2008 protocol. Res Pract Thromb Haemost.

[28] Kuhle S, Spavor M, Massicotte P, Halton J, Cherrick I, Dix D (2008). Prevalence of post-thrombotic syndrome following asymptomatic Thrombosis in survivors of acute lymphoblastic Leukemia. J Thromb Haemost.

[29] Silvis SM, de Sousa DA, Ferro JM, Coutinho JM (2017). Cerebral venous Thrombosis. Nat Rev Neurol.

[30] Kristoffersen ES, Harper CE, Vetvik KG, Zarnovicky S, Hansen JM, Faiz KW (2020). Incidence and mortality of cerebral venous Thrombosis in a Norwegian Population. Stroke.

[31] Couturier MA, Huguet F, Chevallier P, Suarez F, Thomas X, Escoffre-Barbe M (2015). Cerebral venous Thrombosis in adult patients with acute lymphoblastic Leukemia or lymphoblastic Lymphoma during induction chemotherapy with l-asparaginase: the GRAALL experience. Am J Hematol.

[32] Dentali F, Gianni M, Crowther MA, Ageno W (2006). Natural history of cerebral vein Thrombosis: a systematic review. Blood.

[33] Coutinho JM, Zuurbier SM, Stam J (2014). Declining mortality in cerebral venous Thrombosis: a systematic review. Stroke.

[34] Ferro JM, Canhão P, Stam J, Bousser MG, Barinagarrementeria F, Investigators ISCVT (2004). Prognosis of cerebral vein and dural sinus Thrombosis: results of the International Study on Cerebral Vein and Dural Sinus Thrombosis (ISCVT). Stroke.

[35] Zuurbier SM, Lauw MN, Coutinho JM, Majoie CBLM, van der Holt B, Cornelissen JJ (2015). Clinical course of cerebral venous Thrombosis in adult Acute Lymphoblastic Leukemia. J Stroke Cerebrovasc Dis off J Natl Stroke Assoc.

[36] Wani NA, Kosar T, Pala NA, Qureshi UA (2010). Sagittal sinus Thrombosis due to L-asparaginase. J Pediatr Neurosci.

[37] van der Sluis IM, Vrooman LM, Pieters R, Baruchel A, Escherich G, Goulden N (2016). Consensus expert recommendations for identification and management of asparaginase hypersensitivity and silent inactivation. Haematologica.

[38] Skipper MT, Rank CU, Jarvis KB, Lynggaard LS, Andrés-Jensen L, Quist-Paulsen P (2022). Cerebral sinovenous Thrombosis and asparaginase re-exposure in patients aged 1–45 years with acute lymphoblastic Leukaemia: a NOPHO ALL2008 study. EJHaem.

[39] Zwicker JI, Wang TF, DeAngelo DJ, Lauw MN, Connors JM, Falanga A (2020). The prevention and management of asparaginase-related venous thromboembolism in adults: Guidance from the SSC on Hemostasis and Malignancy of the ISTH. J Thromb Haemost JTH.

[40] Napolitano M, Saccullo G, Marietta M, Carpenedo M, Castaman G, Cerchiara E (2019). Platelet cut-off for anticoagulant therapy in thrombocytopenic patients with blood cancer and venous thromboembolism: an expert consensus. Blood Transfus Trasfus Sangue.

[41] Durrani J, Malik F, Ali N, Jafri SIM (2018). To be or not to be a case of heparin resistance. J Community Hosp Intern Med Perspect.

[42] Polska Grupa ds. Leczenia Białaczek u Dorosłych. Protokół leczenia ostrej białaczki limfoblastycznej u dorosłych. Polish Adult Leukemia Group (PALG) guidelines for the treatment of ALL. [Internet]. 2018 [cited 2023 Jul 16]. Available from: www.palg.pl.

[43] Anderson DR, Stock W, Karrison TG, Leader A (2022). D-dimer and risk for Thrombosis in adults with newly diagnosed acute lymphoblastic Leukemia. Blood Adv.

