# Supplementary material for: Superior sagittal sinus thrombosis in the course of mixed phenotype acute leukaemia treated with acute lymphoblastic leukaemia-like therapy—a case report
Source: Thromb J. 2023 Nov 16;21:117. doi: 10.1186/s12959-023-00561-9 (PMC10652634; doi:10.1186/s12959-023-00561-9)
Supplement: Supplementary file 1 — Supplementary Material 1 [file 12959_2023_561_MOESM1_ESM.docx]

**Supplementary Table 1.** European Group of Immunological Classification of Leukemia scoring system. Derived from Bene et al., Leukemia 1995 (6).

|  | **B lineage** | **T Lineage** | **Myeloid Lineage** |
| --- | --- | --- | --- |
| **0.5 Points** | TdT  CD24 | TdT  CD1a  CD7 | CD14  CD15  CD64  CD117 |
| **1 Point** | CD19  CD10  CD20 | CD2  CD5  CD8  CD10 | CD13  CD33  CDw65 |
| **2 Points** | CD79a  cyt IgM  cyt CD22 | CD3 (cytoplasmic/surface)  anti-TCR α/β  anti-TCR γ/δ | anti-MPO  anti-lysozyme |

Underlined are those, which are actual in our case.
